# Impact of aesthetic restorative treatment on anterior teeth with fluorosis among residents of an endemic area in Brazil: intervention study

**DOI:** 10.1186/1472-6831-14-52

**Published:** 2014-05-13

**Authors:** Thalita Thyrza De Almeida Santa-Rosa, Raquel Conceição Ferreira, Andréia Maria Araújo Drummond, Cláudia Silami De Magalhães, Andréa Maria Duarte Vargas, Efigênia Ferreira E Ferreira

**Affiliations:** 1 Department of Dentistry, State University of Montes Claros, Montes Claros, MG, Brazil; 2 Department of Community and Preventive Dentistry, School of Dentistry, Federal University of Minas Gerais, Belo Horizonte, MG, Brazil; 3 Postgradute Program in Dentistry, Department of Community and Preventive Dentistry, School of Dentistry, Federal University of Minas Gerais, Belo Horizonte, MG, Brazil; 4 Department of Restorative Dentistry, School of Dentistry, Federal University of Minas Gerais, Belo Horizonte, MG, Brazil

**Keywords:** Fluorosis, Dental, Endemic diseases, OHIP-14

## Abstract

**Background:**

Endemic dental fluorosis has already been described in some regions of the world. The aim of this study was to evaluate the functional and psychosocial impact of direct aesthetic restorative treatments in endemic fluorosis patients in the northern state of Minas Gerais, Brazil. Was a quasi-experimental intervention study.

**Methods:**

The reference population consisted of individuals between 9 and 27 years of age that were served by a project intended to recover the smiles of patients with severe fluorosis. The questionnaires were administered on two occasions, 24 months apart (before and after dental treatment). Initially, descriptive analyses were conducted. Prevalence and severity, as well as the extent of the functional and psychosocial impact of oral disorders were estimated based on the Oral Health Impact Profile instrument (OHIP-14). Comparisons between baseline and follow-up and between treatment techniques were carried out using the McNemar, Wilcoxon, and Mann–Whitney tests.

**Results:**

The study involved 53 individuals, with a mean age of 15.9 years, treated with microabrasion, dental composite, or a combination of both techniques. The treatments performed proved to be competent for reducing the functional and psychosocial impact of oral disorders as measured by the OHIP-14, pointing to the possibility of establishing protocols to be used in programs aimed at restoring the aesthetics and functionality of the anterior teeth in large populations.

**Conclusions:**

After performing the direct aesthetic restorative treatments in patients with endemic fluorosis, a significant improvement was observed in the prevalence and severity, as well as the extent of the functional and psychosocial impact of oral disorders.

## Background

Dental fluorosis is a change in the enamel that results from exposure to excessive and continued fluoride intake during tooth formation. Its degree of manifestation depends on the fluoride dose ingested, time, and exposure duration, as well as each individual’s response [1], but a significant dose–response relationship has been observed [2]. Clinically, dental fluorosis is characterized by opaque enamel, with color patches that may range from white to dark brown, or in more severe stages, areas of hypoplasia and erosion [3-5].

Fluoridation of the public water supply is one of the key measures aimed at reducing the levels of caries in the population [6,7]. The minimum and maximums desirable levels of fluoride in public water supplies have been defined by the World Health Organization (concentrations between 0.9 and 1.2 mg/l). However, in some regions of the world, because of the soil, tests of the water supply have indicated high levels of naturally occurring fluoride that causes the serious problem of dental fluorosis in populations, a phenomenon that was reported in several regions across the globe [8]. In Brazil, moderate and severe endemic dental fluorosis (with a clinically visible loss of tooth structure) has already been described in some estates, such as Ceará, Minas Gerais, Paraíba, São Paulo and Santa Catarina [9-13].

In the northern region of the state of Minas Gerais, Brazil, the 1980s water supply shortage in the rural communities resulted in a demand for deep tube wells as the only alternative during droughts. However, no fluoride testing was done on that water. Then, in the mid-1990s when the first cases were observed, a severe fluorosis situation was already in place, with a high aesthetic and functional tooth impairment in children and adolescents, a phenomenon that became locally known as “rusty tooth” [14].

Given these facts, scientific investigations were conducted in the region to establish a diagnosis for the problem, and average fluoride levels were between 3 and 4 mg/L [13], 4.6 times greater than that of the value indicated by the relevant Brazilian legislation [15]. In an epidemiological study conducted in seven rural communities in this area with a population ranging in age from 6 to 22 years, the prevalence of dental fluorosis was 80.4% and of severe dental fluorosis was 48.9% [16,17].

The observed fluorosis resulted in generations stigmatized by deformities of the tooth surfaces, negatively impacting the quality of life and health of these people [14,18]. In 2007, a project began to help predict, among its activities, the performance of aesthetic restorative treatment on the anterior teeth, which was affected by fluorosis and intended to help the social lives of children and adolescents by recovering their smiles. These children and adolescents were perceived to be “dirty” and careless; therefore, these children and adolescents did not usually smile, and if they did, they hid their teeth with their hands [14].

In dentistry many indicators have been used to evaluate the impact of oral health on the quality of life, including the Oral Health Impact Profile (OHIP). However, OHIP measures the frequency with which functional and psychosocial impacts associated with the oral disorders are experienced, not explicitly address the issue of quality of life [19-22].

Thus, this study aimed to assess the impact of aesthetic restorative treatment (direct resin veneers or microabrasion) on the anterior teeth in patients with endemic fluorosis living in the northern region of the state of Minas Gerais, Brazil.

## Methods

### Design (Study Area and Sample)

This was a quasi-experimental intervention study. The study was conducted in the rural area of the municipality of São Francisco, a semiarid region of the state that is supplied with water collected in deep tube wells. Of the 53,828 inhabitants, 36.5% live in rural areas, and 19.7% of the households consume water from tube wells. It is a poor area with few jobs and scarce access to goods and services. It has an average per capita household monthly income of US$133.00 and a Human Development Index (HDI) of 0.68. The HDI is composed of indicators of health, education and income, ranging from zero (lowest value) to a (higher value). The municipality is situated in the range of Medium Human Development Index (HDI between 0.6 and 0.699) [23].

The reference population consisted of individuals who had dental fluorosis with an aesthetic impairment. Initially, subjects with fluorosis and a Thylstrup and Fejerskov index (TFI) of ≥5 were given preference. The TFI classifies dental fluorosis based on clinical appearance with scores ranging from zero (enamel presents the normal translucency) to nine (loss of main part of enamel with change in anatomic appearance of surface). Scores of 5 or more denote increasing degrees of loss of outermost enamel [3]. Later, subjects with fluorosis and a TFI <5 were also included, given the subjective nature of aesthetic perception. Planning for the restorative procedures only included the restoration of the anterior teeth primarily because of two reasons: these were the prime targets of smile restoration, and these were the procedures that could actually be performed.

Restorative treatment began in 2009 and was performed by a dental surgeon previously trained according to specific protocols for building direct resin veneers [24] and microabrasion [25], intended to reduce any possible bias. The direct technique was chosen because it was feasible to be performed in the individuals’ homes, using mobile equipment. The affected posterior teeth would require prosthetic restoration, which was not covered by these services and impractical for the researchers in terms of time, travel, and available resources. Patients requiring treatments other than the ones proposed by the project were referred to the municipal health network.

The subjects were informed of the existence of the smile restoration project through posters, flyers, local radio news, and lectures in schools. Additionally, the cooperation of the community health workers was instrumental. These professionals previously participated in training sessions that included information on the causes and dental manifestations of fluorosis.

The timing and scheduling for the visits was provided. The restorative dental treatments performed on these patients were microabrasion of the dental enamel, direct aesthetic veneers of the composite resin, or a combination of microabrasion and veneers, according to the severity of the case. The greater the loss of tooth structure and aesthetic impairment, the greater the indication of direct veneers. Participants who had several teeth treated and received veneers or a combination of microabrasion and veneers were included in the group with the highest score (direct veneers).

This study followed the standards and guidelines of the Resolution 466/2012 of the National Health Council that regulates research involving human subjects and was approved by the Ethics Committee of the Federal University of Minas Gerais (UFMG) under opinion no. 260/06.

### Measurements and analysis

In this study, two trained researchers interviewed the participants on two occasions, 24 months apart. The first interview (baseline) was performed during the completion of the patients’ dental records (prior to the aesthetic restorative treatment). The second interview (follow-up) was performed 24 months after baseline and was conducted in the homes of the participants, whether living in an urban or a rural area. Socio-demographic information (gender, date of birth, nationality, occupation, and address) and the initiated treatment (treated tooth, treatment type, date, color choice, and brand of resin used) were collected in the dental records.

In order to assess the functional and psychosocial impact of fluorosis, as well as the intervention, the short version of the Oral Health Impact Profile (OHIP-14) instrument was used [26] that is tested and validated for the Portuguese language [27]. This instrument consists of seven conceptual dimensions (functional limitation, pain, psychological discomfort, physical inability, psychological inability, social inability, and disability) that are formulated with two items in each dimension. The questions have five answer choices, according to the Likert Scale: never (0), rarely (1) sometimes (2), often (3), and always (4). It was requested that the answers be based on experiences from the last 12 months [26].

A descriptive analysis of the results was performed, and the frequency of responses obtained (always, often, sometimes, rarely, and never) for each item in the instrument at two times: baseline and follow-up. The *always/often* and *rarely/never* categories were aggregated.

The prevalence, extent and severity of the functional and psychosocial impact of oral disorders (fluorosis) were estimated, as suggested by Slade and colleagues [28] at both times. To estimate prevalence of the impact, the frequency of interviewees who answered *always/often* to one or more questions on the OHIP-14 was calculated. Participants who answered always or often to one or more questions on the OHIP-14 were considered with impact. The extent of the impact was evaluated by calculating the sum of the number of items denoting impact (*always/often* answers), ranging from 0 to 14. On the other hand, the severity was estimated by adding the codes assigned to each item that resulted in scores ranging from 0 to 56 points, with the highest values corresponding to the greatest functional and psychosocial impact of oral disorders [26].

The effect of the intervention, specifically, the aesthetic restorative treatment, as perceived by the subjects, was verified by comparing the prevalence of participants who had distinguished an impact between both times using McNemar’s test. The Wilcoxon test was used to compare the severity of impact between baseline and follow-up*,* considering the total OHIP-14 score and the score for each dimension. These comparisons were also performed separately considering those participants whose treatment was microabrasion, composite resin, or a combination of both methods. These last two formed a single group as the most invasive intervention (composite resin) was indicated during the restorative planning for both of these treatments. The Wilcoxon test was also used to compare the extent of the impact between baseline and follow-up*.*

The Mann–Whitney test was used to compare the severity of the impact among participants undergoing different types of aesthetic restorative treatments at both evaluation times. The Statistical Package for the Social Sciences software (SPSS, version 17.0) was used for analysis of the data.

## Results and discussion

### Results

There were 57 individuals who participated in this study, of which 50.9% were male. The mean age of the participants was 15.9 years (±4.8 years; 9–27 years), and the median age was 16 years. Regarding occupation, most were students (77.2%), followed by rural workers (14%), domestic workers (3.5%), merchants, housewives, and teachers (1.8% each).

Fifteen participants were lost to follow-up. Most dropped out because they left the city to find jobs (according to their family members), and two individuals refused to continue as participants in the study.

**Figure 1:**
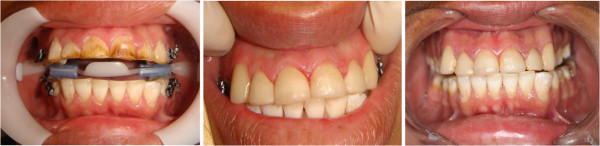
Teeth with severe fluorosis treated with direct resin aesthetic veneers (baseline, after treatment, and follow-up).

As for the aesthetic restorative dental treatment (Figure 1), most of the participants had microabrasion of the dental enamel (70.2%), while 14.0% of the participants had direct aesthetic composite resin veneers and 15.8% had a combination of microabrasion and veneers.

At baseline, most participants reported *never* for 11 of the 14 OHIP-14 items. Approximately one-third of the participants (33.3%) reported always or often feeling “worried about their teeth”, and 17.5% reported being “ashamed of their teeth”. At follow-up, there was a reduction in the frequency of participants who reported always or often feeling “worried about their teeth” (9.5%) and feeling “ashamed of their teeth” (7.1%). Taking into account all items of the OHIP-14, the prevalence of the functional and psychosocial impact on oral disorders was 43.9% and 11.9%, at baseline and follow-up, respectively (Table 1).

**Table 1:** Impact frequency reported in each dimension of the OHIP-14 among study participants at baseline and upon follow-up, São Francisco, Brazil

**OHIP-14**		**Never/Rarely**				**Sometimes**				**Often/Always**			
		**Baseline**		Follow-up*		**Baseline**		Follow-up*		**Baseline**		Follow-up*	
		**n**	**%**	**n**	**%**	**n**	**%**	**n**	**%**	**n**	**%**	**n**	**%**
**Functional limitation**	1 Have you ever had problems speaking a word because of your teeth?	54	94.7	39	92.9	3	5.3	3	7.1	0	0	0	0
	2 Did the taste of food become worse because of your teeth?	49	86.0	40	95.2	8	14.0	2	4.8	0	0	0	0
**Physical pain**	3 Did you feel pain in your teeth?	16	28.1	23	54.8	39	68.4	19	45.2	2	3.5	0	0
	4 Did you have any trouble eating food because of your teeth?	38	66.7	28	66.7	17	29.8	13	31	2	3.5	1	2.4
**Psychological discomfort**	5 Were you worried because of your teeth?	14	24.6	17	40.5	24	42.1	21	50	19	33.3	4	9.5
	6 Did you feel stressed (nervous) because of your teeth?	37	64.9	30	71.4	17	29.8	12	28.6	3	5.3	0	0
**Physical disability**	7 Did you have trouble eating because of your teeth?	44	77.2	32	76.2	12	21.1	9	21.4	1	1.8	1	2.4
	8 Did you have to stop eating because of your teeth?	52	91.2	38	90.5	5	8.8	3	7.1	0	0	1	2.4
**Psychological disability**	9 Did you have trouble relaxing (staying calm) because of your teeth?	46	80.7	34	81	11	19.3	8	19	0	0	0	0
	10 Did you feel ashamed of your teeth?	17	29.8	28	66.7	30	52.6	11	26.2	10	17.5	3	7.1
**Social disability**	11 Did you get angry with others because of your teeth?	35	61.4	33	78.6	18	31.6	9	21.4	4	7	0	0
	12 Did you have trouble performing your work and daily activities because of your teeth?	53	93	40	95.2	2	3.5	2	4.8	2	3.5	0	0
**Handicap**	13 Did you feel that life had worsened because of your teeth?	38	66.7	37	88.1	16	28.1	4	9.5	3	5.3	1	2.4
	14 Were you unable to do the things you normally do every day, because of your teeth?	51	89.5	41	97.6	4	7	1	2.4	2	3.5	0	0
			No impact (%)					With impact (%)					
			Baseline			Follow-up		Baseline			Follow-up		
Functional and psychosocial impact of oral disorders – Taking into account all itens of the OHIP-14			56.1			88.1		43.9			11.9		

*15 participants were lost to follow-up.

Table 2 depicts the prevalence changes observed between baseline and follow-up, highlighting the migration of participants from “no impact” conditions (sometimes, rarely, or never) to “with impact” (always or often) conditions. Of the 15 participants who had an impact at baseline, 11 had no impact after the aesthetic restorative treatment (p = 0.006). Considering the prevalence of functional and psychosocial impact according to the OHIP-14 dimensions, there was a significant reduction in the prevalence of impact on the psychological discomfort dimension (Table 3). While considering the groups treated with microabrasion, composite resin, or composite resin/microabrasion, a comparison of the prevalence of the impact at the two separate times showed a significant reduction in the frequency of subjects who had an impact among those treated with resin (p = 0.031). There was no significant reduction in the group treated with microabrasion (p = 0.219).

**Table 2:** Participants with and without functional and psychosocial impact of oral disorders at baseline and follow-up, São Francisco, Brazil

		***Follow-up***		
		**No impact**	**With impact**	**Total**
*Baseline*	No impact	26	1	27
	With impact	11	4	15
	Total	37	5	42
	p = 0,006 (McNemar’s test)			

**Table 3:** Comparison of the frequency of participants with an impact at baseline and no impact upon follow-up according to the OHIP-14 dimensions, São Francisco, Brazil

**OHIP-14 dimensions**	Baseline* **(n = 57) n (%) with impact**	***Follow-up *** (n = 42)* n (%)			p-value***
		**No impact**	With impact**	Losses**	
Functional limitation	0	0	0	0	-
Physical pain	4 (7.0)	3 (7.0)	0	1 (2.4)	0.625
Psychological discomfort	19 (33.3)	8 (19.1)	3 (7.1)	8 (19.1)	0.039
Physical disability	1 (1.8)	1 (2.4)	0	0	1.00
Psychological disability	10 (17.5)	5 (11.9)	1 (2.4)	4 (9.5)	0.453
Social disability	5 (8.8)	3 (7.1)	0	2 (4.8)	0.250
Handicap	3 (5.3)	1 (2.4)	1 (2.4)	1 (2.4)	1.00

*Individuals with no impact at baseline were not presented.

**The frequencies of individuals with no impact and losses upon follow-up were included to highlight the migration of responses.

***p-value assessed by McNemar’s test.

Regarding severity (sum of all scores), the mean OHIP-14 value found at baseline was 9.8 (±6.7), with a minimum of zero, a maximum of 32, and a median of 10. Upon follow-up*,* the mean OHIP-14 value was 5.9 (±5.5), with a minimum of zero, a maximum of 20, and a median of 4. There was a significant reduction in OHIP-14 scores at the follow-up for the total sample and in the group of participants whose were treated with microabrasion. At both times, the greatest impact was observed among participants in the group whose treatment was composite resin or composite resin and microabrasion combination (Table 4). A comparison of the severity OHIP-14 scores by dimension between the two times showed a significant reduction for the domains psychological discomfort (p = 0.04), psychological disability (p = 0.009), and handicap (p = 0.008) at the follow-up.

**Table 4:** Comparisons of the severity of impact (sum of all scores), at the two times in both restorative treatment groups, São Francisco, Brazil

	***Total sample***	***Treatment***		***p-value (Mann–Whitney test)***
		**Microabrasion**	**Composite resin; Combination of microabrasion and composite resin**	
*Baseline*	10 (5)*	8.0 (6.0)*	10 (8.0)*	0.002
*Follow-up*	4 (8.0)*	3.0 (8.0)*	8 (8.0)*	0.015
*p*-value (Wilcoxon test)	0.003	0.007	0.180	

*Values refer to medians and interquartile distances.

**Figure 2:**
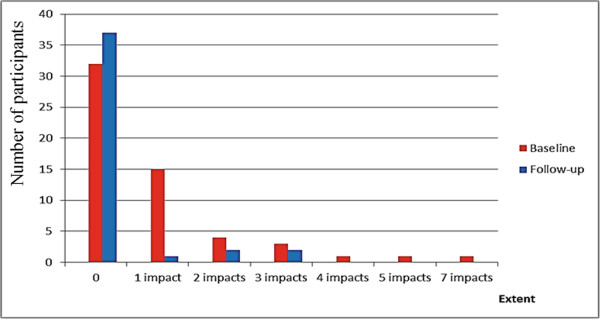
**Extent of impact (number of items with *always/often *** **answers) at baseline and upon follow-up, São Francisco, Brazil.**

Regarding the extent of the impact (number of items with *always/often* answers), the average at baseline was 0.82 and upon follow-up was 0.26 (p = 0.05, Wilcoxon test). At baseline, 15 (26.3%) of the participants had one impact, 4 (7.0%) had two impacts, and 6 (10.7%) had three or more impacts (3 had three impacts, 1 had four, 1 had five and 1 had seven impacts); the frequencies for the number of impacts upon follow-up were: one impact = 1 (2.4%), two impact = 2 (4.8%), and three impact = 2 (4.8%). The maximum number of impacts upon follow-up was three (Figure 2).

### Discussion

The study site was a region of Minas Gerais with a history of a high prevalence and severity of endemic fluorosis caused by natural fluoride in the water supply of the rural communities [16-18]. The participants in the study were residents of this region, and most of them were students, with a mean age of 15.9 years, thus, during adolescence [29].

This study showed that two years after the performance of the direct aesthetic restorative treatments in patients with endemic fluorosis, significant improvements were noted in the prevalence, severity and extent of functional and psychosocial impact of the oral disorders, as measured by the OHIP-14. As dental fluorosis in its moderate or severe forms causes functional and aesthetic changes that interfere with personality development and integration in the labor market [10], the participants’ reports of concern and embarrassment because of their teeth at the initiation of the study are concerning. The significant reduction in the prevalence of the functional and psychosocial impact of oral disorders following direct restorative dental treatment reinforces previous findings from studies on fluorosis pertaining to the patients’ dissatisfaction with their appearance, low self-esteem, and feelings of social exclusion [10,18,30].

The direct dental treatments (which eliminate the need for dental prosthetic laboratories) were chosen because of their lower cost, greater preservation of the healthy tooth structure, and good aesthetics. This option opposes the hegemonic idea in dentistry that traditionally associates quality with more sophisticated treatments, which are causing financial barriers to access dental care. Moreover, it helps achieve the ethical premise of balance between “good aesthetics” and avoiding biological damage over the long run [31]. Microabrasion of the dental enamel, a procedure performed on most of the participants’ teeth, is a simple and low-cost technique [32].

After having the restorative treatments for 24 months, the participants experienced a reduction in the prevalence of the functional and psychosocial impact of oral disorders from 43.9% to 11.9%. The higher frequency of “always” or “often” responses for the dimensions of discomfort and psychological disability at baseline and upon follow-up indicates the chronic (non-transient) character of the psychosocial impact of oral disorders on the participants’ lives [22,28]. A study by Castilho and colleagues [18] found that students affected with fluorosis felt embarrassed to smile at strangers, because of an apparent association between fluorosis and a lack of dental hygiene. Findings from the study included conflicts between affected and unaffected students, problems pursuing a romantic relationship, and uncertainty regarding a future career. The severe dental fluorosis injuries appeared to be a stigmatizing factor and contributed to the exclusion of an entire generation of adolescents and young people by negatively impacting the quality of life and health of these people. Restorative treatment seems to have contributed towards a significant reduction in impact among the participants. Only one participant migrated from the “no impact” condition to the “with impact” condition in the period between treatment completion and follow-up*.* At the time of assessment, this participant reported not visiting a dentist for a long time and considered his own teeth dark, requiring bleaching. After 24 months, the effect of the treatment did not satisfy him any longer.

A reduction in the prevalence of impact only among those subjects treated with resin can be related to the issue of expectations generated by the treatment. Patients in need of composite resin or composite resin combined with microabrasion clinically demonstrated a greater aesthetic impairment prior to treatment. It is believed that these individuals have incurred more embarrassment than those whose treatment only included microabrasion, and their satisfaction with the end result had a greater impact (easily achieved). Studies by Castilho and colleagues [14,18] reported that the desire of students with fluorosis to receive dental treatment improved their social relationships, thus reinforcing the hypothesis regarding the anticipation of treatment. Such reports support the significant reduction in the number of participants experiencing an impact on the dimension of psychological discomfort.

When the severity of impact was estimated, there was a significant reduction in the overall OHIP-14 score between baseline and follow-up in the dimensions of psychological discomfort, psychological disability, and handicap. These dimensions include emotional behavior, difficulty relaxing, feelings of shame, and disadvantages in daily life. By separating the study participants into two groups according to the treatment received, a significant reduction in severity was detected in the microabrasion group; however, the greatest impacts were still observed among participants in the group whose treatment was composite resin or composite resin combined with microabrasion at both times.

A significant reduction in the overall OHIP-14 score can be interpreted as an improvement in the functional impact, psychosocial impact, or both on the oral health in the lives of participants, considering that it denotes a change in the chronic nature of this impact [22].

During the interviews, the OHIP-14 showed to be quick and easy application and good understanding by respondents. The OHIP-14 was used in this study, seeking information regarding the functional and psychosocial impact of the oral disorders before and after restorative treatment by estimating the prevalence, severity, and extent of this impact, as recommended by Slade and colleagues [28]. Therefore, no attempt was made to assess the impact of the oral health condition on the quality of life based on the OHIP-14, a question already well discussed by Locker and Quiñonez [22].

The two-year follow-up evaluation was deliberate, as the visual impact is large and proportional to satisfaction immediately after the aesthetic restoration through any type of treatment [33].

### Strengths and limitations

Study participants had long been yearning for restorative dental treatment that might bring back their ability to smile, even at strangers, thus facilitating social inclusion. Moreover, smiling is a universal act of human behavior, common to all cultures and is “a ritual of approach” [34]. The treatments performed proved to be competent for reducing the functional and psychosocial impact of the oral disorders as measured by the OHIP-14, pointing to the possibility of establishing protocols to be used in programs aimed at restoring the aesthetics and functionality of the anterior teeth in large populations.

Besides the importance of dental intervention, it should be noted that another contribution of this study was the training of the community health workers regarding the causes and dental manifestations of fluorosis. Considering that this profession came into existence with the core idea of serving as a link between the community and the public health system [35], as well as acting as multipliers of information, it was essential to clarify that the dental changes were a result of the excessive and continued ingestion of fluoride and not of a lack of personal hygiene (self-care) as many thought.

Because of ethical issues, the study had no comparison group; therefore, it cannot be assumed with certainty that the impact was secondary to the treatment. The sample size and the losses may have influenced the analysis, due to loss of statistical power in hypothesis testing. Despite these limitations, the results showed the importance of restorative intervention in decrease the prevalence, severity and extent of the functional and psychosocial impact of oral disorders. It is essential to highlight that the originality of this study complied with the ethical limits of research involving humans.

## Conclusions

Two years after the performance of the direct aesthetic restorative treatments in patients with endemic fluorosis, significant improvements were noted in the prevalence and severity, as well as the extent of the functional and psychosocial impact of the oral disorders, as measured by the OHIP-14.

## Competing interest

The authors declare no competing interest.

## Authors’ contributions

TTASR was responsible for the acquisition of the data, the analysis and interpretation of the data and the organization and drafting of the paper. RCF was responsible for data analysis and interpretation. AMAD and CSM was responsible for acquisition and data interpretation. AMDV and EFF were responsible for the study supervision during data collection and assisted with the data analysis and interpretation, thus contributing critically to the progress of the study. All authors reading and approving the final manuscript.

## Pre-publication history

The pre-publication history for this paper can be accessed here:

http://www.biomedcentral.com/1472-6831/14/52/prepub
